# Intestinal Colonization of IL-2 Deficient Mice with Non-Colitogenic *B. vulgatus* Prevents DC Maturation and T-Cell Polarization

**DOI:** 10.1371/journal.pone.0002376

**Published:** 2008-06-11

**Authors:** Martina Müller, Kerstin Fink, Julia Geisel, Frauke Kahl, Burghardt Jilge, Jörg Reimann, Nicolas Mach, Ingo B. Autenrieth, Julia S. Frick

**Affiliations:** 1 Institute of Medical Microbiology and Hygiene, University of Tübingen, Tübingen, Germany; 2 University of Ulm, Ulm, Germany; 3 Oncology Division, Geneva University Hospital, Geneva, Switzerland; University of Sheffield, United Kingdom

## Abstract

**Background:**

*IL-2* deficient (*IL-2^−/−^*) mice mono-colonized with *E. coli* mpk develop colitis whereas *IL-2^−/−^*-mice mono-colonized with *B. vulgatus* mpk do not and are even protected from *E. coli* mpk induced colitis.

**Methodology/Principal Findings:**

We investigated if mono-colonization with *E. coli* mpk or *B. vulgatus* mpk differentially modulates distribution, activation and maturation of intestinal lamina propria (LP) dendritic cells (DC). LP DC in mice mono-colonized with protective *B. vulgatus* mpk or co-colonized with *E. coli* mpk/*B. vulgatus* mpk featured a semi-mature LP DC phenotype (CD40^lo^CD80^lo^MHC-II^hi^) whereas mono-colonization with colitogenic *E. coli* mpk induced LP DC activation and maturation prior to onset of colitis. Accordingly, chemokine receptor (CCR) 7 surface expression was more strikingly enhanced in mesenteric lymph node DC from *E. coli* mpk than *B. vulgatus* mpk mono- or co-colonized mice. Mature but not semi-mature LP DC promoted Th1 polarization. As *B. vulgatus* mpk promotes differentiation of semi-mature DC presumably by IL-6, mRNA and protein expression of IL-6 was investigated in LP DC. The data demonstrated that IL-6 mRNA and protein was increased in LP DC of *B. vulgatus* mpk as compared to *E. coli* mpk mono-colonized *IL-2^−/−^*-mice. The *B. vulgatus* mpk mediated suppression of CCR7 expression and DC migration was abolished in *IL-6^−/−^*-DC *in vitro*.

**Conclusions/Significance:**

From this data we conclude that the *B. vulgatus* triggered IL-6 secretion by LP DC in absence of proinflammatory cytokines such as IL-12 or TNF-α induces a semi-mature LP DC phenotype, which might prevent T-cell activation and thereby the induction of colitis in *IL-2^−/−^-*mice. The data provide new evidence that IL-6 might act as an immune regulatory cytokine in the mucosa by targeting intestinal DC.

## Introduction

The current pathophysiologic concept of inflammatory bowel diseases (IBD) proposes a multifactorial event with environmental, immunologic and genetic contributory factors [1], [2]. In some animal models environmental factors e.g. the intestinal microbiota, in addition to the genetic background have been identified as crucial factors for the development of IBD. Thus germfree (GF) and *Bacteroides vulgatus* mpk mono-colonized *IL-2^−/−^*-mice remain healthy whereas *IL-2^−/−^*-mice raised in a specific-pathogen-free (SPF) environment or when mono-colonized with *E. coli* mpk develop the disease [3]–[5]. DNA array experiments and cellular studies revealed that *E. coli* mpk is a non-pathogenic commensal *E. coli* strain [3].

DC are antigen presenting cells (APC) with important functions in the mucosal immune system. They have the capacity to stimulate naïve T-cells and thus are thought to be initiators of T-cell responses [6]. Intestinal DC transport microbial antigens to the mesenteric lymph nodes (MLN) and the spleen which causes the induction of systemic immune responses [7]. Activation and maturation of DC is induced by infectious agents and inflammatory products leading to increased expression of co-stimulatory and MHC-II molecules, cytokine production and T-cell activation [8]–[15]. In addition to bacterial stimuli, DC maturation is also regulated by cytokines [16].

Recent studies suggest a suppressive effect of IL-6 on DC activation and maturation [17]–[19]. Splenic DC isolated out of LPS treated *IL-6^−/−^*-mice showed an enhanced expression of CD86, suggesting that IL-6 may act as an immunosuppressive cytokine for DC differentiation [17]. Furthermore *B. vulgatus*-induced semi-mature DC were non-responsive upon restimulation with *E. coli* mpk in terms of DC maturation, T-cell priming and TNF-α production. This effect was abrogated by addition of anti-IL-6 monoclonal antibody (mAb) or mimicked with recombinant IL-6, suggesting that IL-6 induces semi-mature DC. This semi-mature DC were non-responsive to pro-inflammatory activation by *E. coli* mpk [18]. Recently, we described that *N*-palmitoyl-*bis*(palmitoyloxy)-propyl-cysteinylseryl-Lys_4_ (P3C)- or LPS-primed DC were non-responsive to P3C or LPS restimulation in terms of TNF-α production [20]. The mechanisms involved in tolerance were dependent on the concentration of the TLR ligand used for DC priming. Priming with LPS or P3C at high concentrations resulted in DC activation and maturation. In contrast, priming of DC with LPS or P3C at low concentrations resulted in an IL-6-dependent tolerance, which was abolished in IL-6 deficient DC, and was not accompanied by maturation of DC or by downregulation of TLR2 or TLR4 [20]. Together, these data provide evidence that IL-6 might play a bifunctional role in inflammatory diseases. Depending on the host status IL-6 may act as a pro-inflammatory or anti-inflammatory cytokine promoting or inhibiting T-cell priming and Th1 responses.

In this study we investigated whether and how intestinal colonization with *E. coli* mpk or *B. vulgatus* mpk differentially modulates activation, maturation and IL-6 production of intestinal lamina propria (LP) DC in *IL-2^−/−^*-mice prior to onset of colitis and whether differential modulation of LP DC might have an impact on induction or prevention of colitis in gnotobiotic *IL-2^−/−^-*mice *in vivo*.

## Results

### Characterization of LP DC in gnotobiotic *IL-2^−/−^*-mice

*E. coli* mpk induces colitis in *IL-2^−/−^*-mice, whereas the commensal strain *B. vulgatus* does not and even prevents *E. coli* mpk induced colitis [3]. In order to analyze the impact of commensal bacteria on the activation of intestinal inflammatory host reaction prior to onset of colitis we characterized the subsets of intestinal lamina propria (LP) DC, the expression of activation surface marker on LP DC and the host T-cell responses of gnotobiotic *IL-2^−/−^-*mice. Intestinal CD11c^+^CD19^−^CD3ε^−^DX5^−^ LP DC from non-inflamed *B. vulgatus* mpk mono-colonized, non-inflamed *E. coli* mpk*/B. vulgatus* mpk co-colonized *IL-2^−/−^*-mice and non-inflamed *E. coli* mono-colonized *IL-2^−/−^*-mice prone to develop disease were prepared and analyzed by flow cytometry. LP DC from the inflamed intestine of SPF *IL-2^−/−^*-mice and healthy germfree *IL-2^−/−^-*mice served as reference. NK-, T- and B-cells were excluded from the analyses. The analysis of exclusively dendritic cells was ensured by analyzing only CD11c^hi^ positive, lineage negative cells to exclude CD11c^int^ subsets of macrophages.

**Figure 1:**
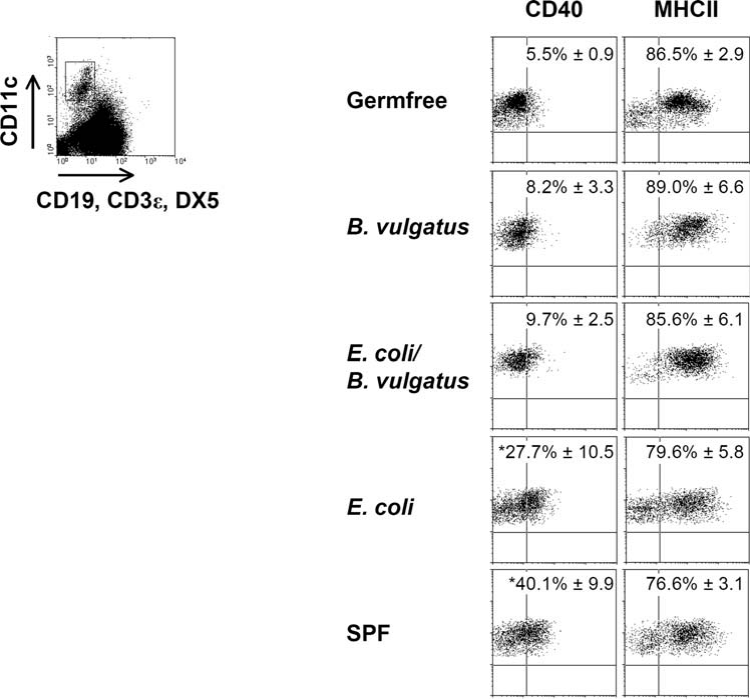
LP DC activation and maturation in germfree, gnotobiotic and SPF mice. LP DC were isolated from the total intestine of GF, *B. vulgatus* mpk or *E. coli* mpk mono-colonized, *E. coli* mpk*/B. vulgatus* mpk co-colonized and SPF *IL-2^−/−^*-mice and analyzed for expression of CD40 and MHC-II by flow cytometry. The values represent at least six animals which were analyzed separately. Mean and SD were calculated from those independent experiments. Numbers indicate the percentage of positive cells±SD. Representative dotplots are shown from each group. * p<0.05 compared to germfree and *B. vulgatus* mpk mono-colonized *IL-2^−/−^*-mice.

The expression of the DC activation marker CD40, CD80 and CD86 on intestinal LP DC was significantly enhanced in *E. coli* mpk mono-colonized mice and SPF mice as compared to *B. vulgatus* mpk mono-colonized, *E. coli* mpk*/B. vulgatus* mpk co-colonized and germfree mice (Figure 1; Figure S1). The phenotype (CD40^lo^CD80^lo^MHC-II^hi^) of LP DC isolated from *B. vulgatus* mpk mono-colonized and from *E. coli* mpk*/B. vulgatus* mpk co-colonized mice matched the semi-mature DC phenotype previously described by us and others [18]–[20]. By contrast, the LP DC of *E. coli* mpk mono-colonized and SPF mice (CD40^hi^CD80^hi^MHC-II^hi^) corresponded to an activated and mature DC phenotype (Figure 1; Figure S1).

**Figure 2:**
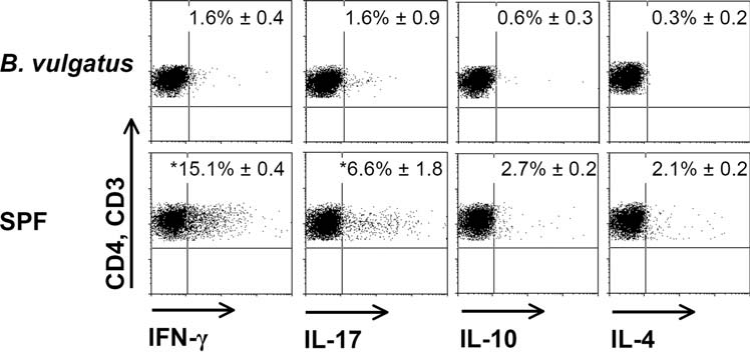
T-cell polarization in *IL-2* *^−^* ^*/−*^-mice prone to develop colitis and *B. vulgatus* mpk mono-colonized mice. CD3^+^CD4^+^ LP T-cells of SPF and *B. vulgatus* mpk mono-colonized *IL-2^−/−^*-mice were prepared, intracellular cytokine staining for IFN-γ, IL-17, IL-4 and IL-10 was performed and the cytokine expression was analyzed by FACS. Numbers indicate the percentage of positive cells±SD. The results are representative for three independent experiments which were analyzed separately. Mean and SD were calculated from those independent experiments.

In germfree or *B. vulgatus* mpk mono- or *E. coli* mpk*/B. vulgatus* mpk co-colonized IL-2^+/+^-wild type (WT) mice the expression of activation and maturation markers on LP DC did not differ from LP DC isolated out of *IL-2^−/−^-*mice except for the expression of MHC class II which was slightly diminished in wild type mice. In *E. coli* mpk mono-colonized and SPF *IL-2^+/+^-*mice the expression of activation markers on LP DC was slightly enhanced as compared to germfree or *B. vulgatus* mpk mono- or *E. coli* mpk*/B. vulgatus* mpk co-colonized wild type mice, however the differences were not significant (Figure S2). Consistent with the mature and activated LP DC from *E. coli* mpk and SPF colonized *IL-2^−/−^-*mice we observed T-cells which were polarized in Th1 direction indicated by increased levels of IFN-γ. In addition, we detected a slight expression of IL-17 in LP T-cells isolated out of SPF *IL-2^−/−^-*mice (Figure 2). Unlike, in *B. vulgatus* mpk mono-colonized mice which do not develop colitis we observed neither IFN-γ or IL-17 nor IL-10 or IL-4 expression indicating that semi-mature LP DC did not lead to T-cell polarization (Figure 2).

We next addressed the question whether the colonization state has an impact on the composition of intestinal lamina propria DC subsets. For this purpose we analyzed the absolute number of LP DC and the percentage of myeloid (CD11c^+^CD11b^+^) and plasmacytoid (CD11c^+^B220^+^) LP DC in the different mouse groups. No differences in the percentage of LP DC subpopulations were observed comparing *E. coli* mpk or *B. vulgatus* mpk mono-colonized, co-colonized or germfree *IL-2^−/−^*-mice. Interestingly, in *IL-2^−/−^* SPF mice already suffering from colitis we observed an increase in plasmacytoid intestinal LP DC (Table 1).

**Table 1:** Absolute number and percentage of total DC, myeloid and plasmacytoid DC.

Colonization	CD11c^+^ cells		Myeloid DC	Plasmacytoid DC
	% of leukocytes	absolute number [×10^5^]	% of CD11c^+^ cells	% of CD11c^+^ cells
Germfree	2.4±0.9	4.08±3.42	75.0±0.8	10.1±3.9
*B. vulgatus*	2.3±0.4	4.02±1.93	71.3±3.8	11.0±4.6
*E. coli/B. vulgatus*	2.3±0.9	3.14±0.69	68.0±6.5	9.4±4.2
*E. coli*	1.1±0.5^a^	2.18±1.16	62.5±12.6	13.1±4.1
SPF	0.8±0.4^a^	3.31±1.85	50.7±8.0^a^	19.9±7.0

a p<0.05 compared to *B. vulgatus* mpk mono-colonized, co-colonized and germfree mice. The results are representative for at least six animals. Numbers indicate the percentage of CD11c positive cells or absolute numbers of CD11c positive cells±SD.

### *B. vulgatus* mpk differentiates DC to a semi-mature phenotype

Recent studies have shown that IL-6 differentiates DC to a semi-mature phenotype (CD40^lo^CD80^lo^MHC-II^hi^) which is characterized by a reduced responsiveness towards LPS amongst others [17]–[20]. In addition, the expression of chemokine receptor (CCR) 7 on DC was downregulated by IL-6 which resulted in reduced DC migration [19].

**Figure 3:**
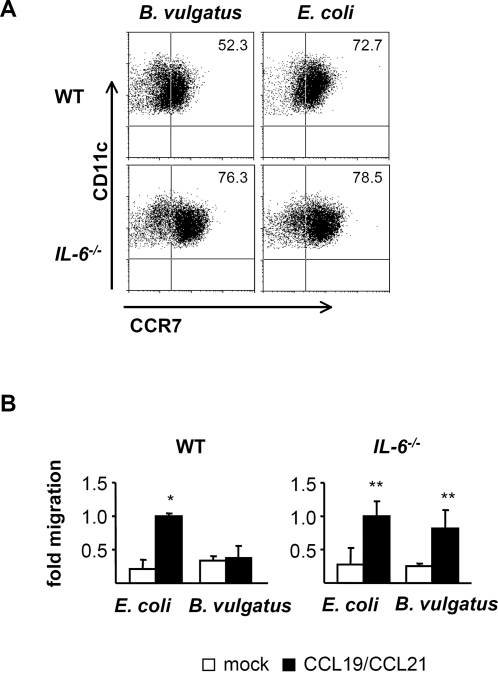
CCR7 expression by and migration of BMDC upon stimulation with *B. vulgatus* mpk or *E. coli* mpk. Analysis of CCR7 surface expression in wild type or *IL-6^−/−^*-BMDC upon stimulation with *B. vulgatus* mpk or *E. coli* mpk (A). CCR7 surface expression was determined by FACS. One representative dotplot is shown out of three independent experiments. *In vitro* chemotaxis of *B. vulgatus* mpk or *E. coli* mpk stimulated wild type or *IL-6^−/−^*-BMDC towards CCL19 and CCL21 (B). 5×10^5^ BMDC were stimulated with *E. coli* mpk or *B. vulgatus* mpk and migration was determined in a 4 h transwell chemotaxis assay. Number of migrated cells of mock controls and *B. vulgatus* mpk primed DC were normalized to the number of *E. coli* mpk stimulated cells migrated along the chemokine gradient to the lower compartment. The results are representative for three independent experiments. Each experiment was performed in triplicates, values represent mean±SD. * p<0.05 compared to mock control and *B. vulgatus* mpk stimulated DC. ** p<0.05 compared to mock control.

**Figure 4:**
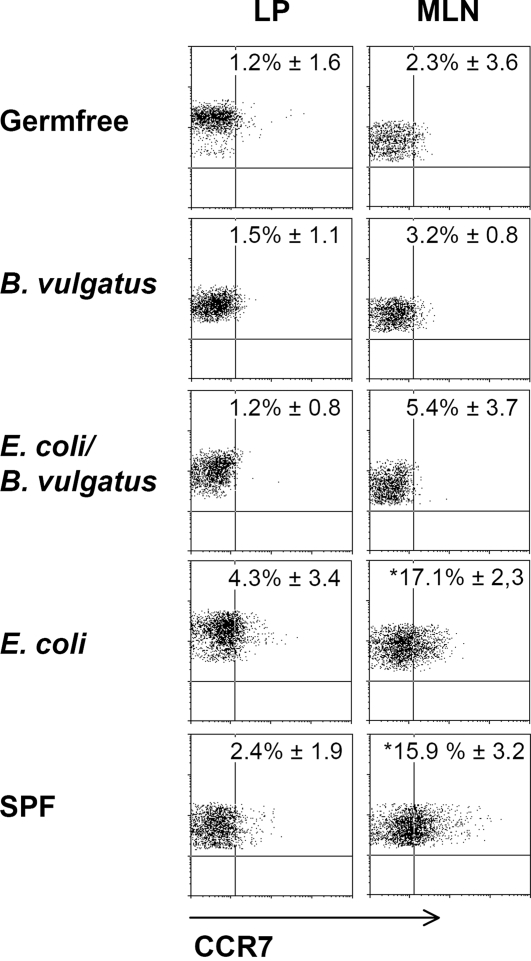
CCR7 expression on LP DC and MLN DC of germfree, gnotobiotic and SPF mice. LP DC or MLN DC were isolated from the total intestine of GF, *B. vulgatus* mpk or *E. coli* mpk mono-colonized, *E. coli* mpk*/B. vulgatus* mpk co-colonized and SPF *IL-2^−/−^*-mice and analyzed for CCR7 expression. The results are representative for at least three animals which were analyzed separately. Mean and SD were calculated from those independent experiments. Numbers indicate the percentage of CCR7 positive cells±SD. * p<0.05 compared to *B. vulgatus* mpk mono-colonized, co-colonized and germfree mice.

Herein we were able to show that mono-colonization of *IL-2^−/−^-*mice with *B. vulgatus* mpk differentiated intestinal LP DC to a semi-mature phenotype and was associated with absence of colitis. We next analyzed the modulation of CCR7 expression and DC migration by priming DC with the protective *B. vulgatus* mpk or the colitogenic *E. coli* mpk. Furthermore the impact of IL-6 on CCR7 expression and migration of DC was analyzed. WT and *IL-6^−/−^-*BMDC were stimulated with *B. vulgatus* mpk or *E. coli* mpk and CCR7 surface expression as well as CCR7 mRNA expression was determined. Priming WT BMDC with *B. vulgatus* mpk led to a significantly reduced CCR7 surface expression as compared to DC primed with *E. coli* mpk. In line with other studies, the *B. vulgatus* mpk primed semi-mature DC were less responsive towards subsequent LPS stimulation (Figure S3). In *IL-6^−/−^*-DC the inhibitory effect of *B. vulgatus* mpk regarding the CCR7 expression and the LPS responsiveness was abolished, indicating that *B. vulgatus* mpk suppresses CCR7 expression in an IL-6 dependent way (Figure 3A; Figure S3 and S4). Accordingly, in a transwell migration assay *B. vulgatus* mpk primed WT DC showed reduced migration towards the CCR7 ligands CCL19 and CCL21 as compared to *E. coli* mpk primed DC. The reduced migration of *B. vulgatus* mpk primed WT DC was abolished in *IL-6^−/−^*-DC. This indicates that the IL-6 dependent expression of CCR7 upon priming with *B. vulgatus* mpk leads to a reduced migration property of DC compared to *E. coli* mpk stimulated DC (Figure 3B). We next analyzed the CCR7 expression *in vivo* on intestinal lamina propria DC and DC isolated from mesenteric lymph nodes (MLN) of *E. coli* mpk or *B. vulgatus* mpk mono-colonized, *E. coli/B. vulgatus* co-colonized, SPF or germfree *IL-2^−/−^*-mice (Figure 4). No significant differences in the examined mouse groups were observed on LP DC (Figure 4). By contrast, DC isolated from the MLN of *E. coli* mpk mono-colonized mice showed a significantly increased expression of CCR7 as compared to *B. vulgatus* mpk mono-colonized mice, *E. coli* mpk*/B. vulgatus* mpk co-colonized or germfree mice. These data might indicate that LP DC of *E. coli* mpk mono-colonized mice are activated and migrate to the MLN while LP DC of *B. vulgatus* mpk mono-colonized mice develop a CD40^lo^CCR7^lo^ phenotype and seem to be less migratory.

In line with these findings, the differential CCR7 expression on LP DC of *E. coli* mpk or *B. vulgatus* mpk mono-colonized mice resulted in different numbers of intestinal lamina propria DC. FACS analysis revealed significantly increased numbers of CD11c^+^ cells in the intestine of germfree, *B. vulgatus* mpk mono-colonized and *E. coli* mpk */B. vulgatus* mpk co-colonized mice as compared to *E. coli* mpk mono-colonized and SPF mice (Table 1). We couldn't observe any differences in the location of CD11c^+^ cells in the lamina propria (Figure S5).

### *B. vulgatus* mpk primed DC are one source of intestinal IL-6

**Figure 5:**
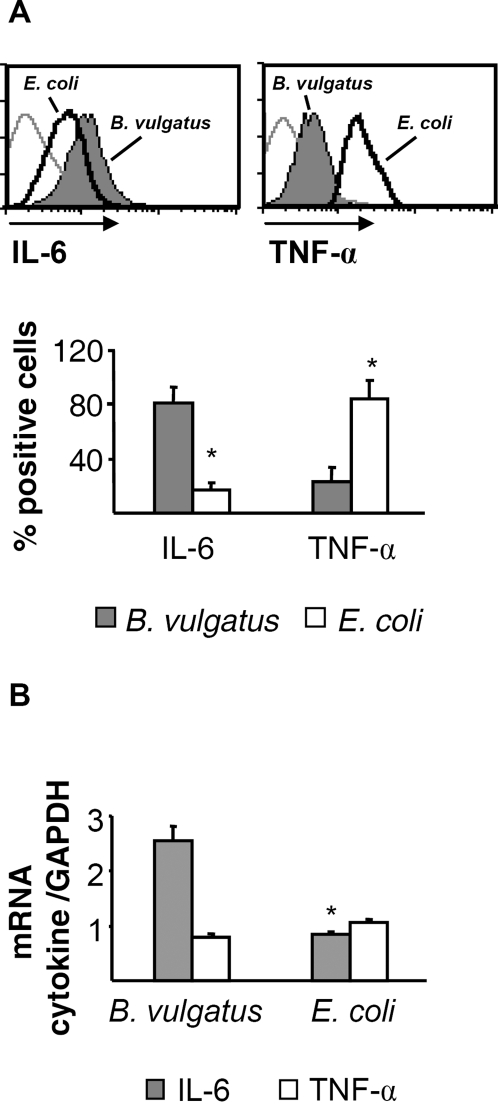
IL-6 and TNF-α expression in LP DC of *E. coli* mpk or *B. vulgatus* mpk mono-colonized mice. LP DC were prepared, intracellular cytokine staining was performed and the cytokine expression was analyzed by FACS (A). For analysis of cytokine mRNA expression, LP DC were enriched by FACS sorting and expression of IL-6 and TNF-α mRNA was analyzed by quantitative RT-PCR (B). * p<0.05 compared to *B. vulgatus* mpk mono-colonized mice.

Recently, we reported that mono-colonization of *IL-2^−/−^*-mice with protective *B. vulgatus* mpk leads to enhanced levels of intestinal IL-6 mRNA and decreased levels of TNF-α mRNA expression as compared to *E. coli* mpk mono-colonized *IL-2^−/−^*-mice [18]. This led to the notion that IL-6 in absence of TNF-α or IL-12 might play an immune-regulatory rather than an immune-activating role [18]. In order to determine whether the enhanced IL-6 level in the intestine of *B. vulgatus* mono-colonized mice originate from intestinal lamina propria DC, we determined the expression of IL-6 and TNF-α by intracellular FACS staining (Fig. 5A) and quantitative RT-PCR (Figure 5B).

The intracellular staining for IL-6 and TNF-α revealed an increased IL-6 cytokine expression in LP DC of *B. vulgatus* mpk as compared to *E. coli* mpk mono-colonized mice. In contrast, expression of TNF-α was significantly reduced in LP DC of *B. vulgatus* mpk mono-colonized mice (Figure 5A). In line with this, IL-6 mRNA levels were significantly enhanced in LP DC of *B. vulgatus* mpk as compared to *E. coli* mpk mono-colonized mice. TNF-α mRNA levels did not differ between LP DC of *B. vulgatus* mpk and *E. coli* mpk mono-colonized mice probably due to degradation of TNF-α mRNA after translation and might lead to the hypothesis that IL-6 mRNA is detectable over a longer period than TNF-α mRNA (Figure 5B).

Taken together our data indicate that *B. vulgatus* mpk might lead to differentiation of LP DC to a semi-mature phenotype, possibly by an IL-6 dependent mechanism. This mechanism might at least partially account for the prevention of colitis induction in *IL-2^−/−^-*mice and might indicate a new immune-regulatory role for IL-6.

## Discussion

The exclusive presence of *B. vulgatus* mpk in the intestine of *IL-2^−/−^*-mice is generally well tolerated without signs of inflammation, whereas *E. coli* mpk elicits a pronounced inflammatory response [3]. We examined the effect of colonization on LP DC activation and maturation. Mono-colonization of *IL-2^−/−^*-mice with non-colitogenic *B. vulgatus* mpk induced a semi-mature LP DC phenotype (CD40^lo^CD80^lo^MHC-II^hi^) [18], whereas mono-colonization with *E. coli* mpk resulted in activated and maturated DC (CD40^hi^CD80^hi^MHC-II^hi^). In *E. coli* mpk mono-colonized and SPF *IL-2^+/+^-*mice no significant activation of LP DC was observed. The enhanced activation of LP DC in *E. coli* mpk mono-colonized and SPF *IL-2^−/−^-*mice might be the consequence of a defective intestinal homeostasis in *IL-2^−/−^-*mice prone to develop colitis.

In *E. coli* mpk but not *B. vulgatus* mpk mono-colonized *IL-2^−/−^*-mice we found, probably due to the *E. coli* mpk induced DC activation, a diminished number of LP DC in the intestine and an enhanced number of CCR7^+^ DC in the MLN. The enhanced expression of CCR7 and MHC-II expression in germfree *IL-2^−/−^*-mice indicates that even these mice lack intestinal microbiota, alimentary-antigens might lead to a slight activation of the mucosa-associated immune system. The absence of colitis in *B. vulgatus* mpk mono-colonized *IL-2^−/−^*-mice was associated with an enhanced production of IL-6 and a decreased production of TNF-α in LP DC as compared to *E. coli* mpk mono-colonized mice. Taken together these data suggest that *B. vulgatus* mpk induced IL-6 might account at least partially for the immune-regulatory function of *B. vulgatus* mpk and the prevention of colitis in *B. vulgatus* mpk mono-colonized *IL-2^−/−^*-mice.

Colitis development in SPF *IL-2^−/−^*-mice was characterized by a predominant Th1 response. However, we cannot exclude that an additional IL-17 response might also contribute to the intestinal inflammation. In presence of IL-6 and TGF-β T-cells differentiate into IL-17-producing Th17 cells [21], [22]. In previous studies we were able to detect intestinal expression of TGF-β and IL-6 mRNA in mice suffering from colitis [23]. This might be a possible explanation for the induction of an additional Th17 response. In line with previous studies [3], [18], the *B. vulgatus* mpk induced semi-mature DC phenotype, neither led to Th1 or Th17 nor to Th2 polarization. This is in line with recent studies showing that IL-6-STAT-3 mediated processes decreased the intracellular MHC-II αβ dimer, li (invariant chain) and H2-DM on DC and suppressed CD4^+^ T-cell mediated immune responses [24].

IL-6 mediates different responses of the host immune system. Anti-inflammatory as well as pro-inflammatory effects have been reported. Various studies showed that IL-6 plays an important role promoting Th2 polarization [25]. Furthermore, IL-6 upregulates the expression of suppressor of cytokine signaling (SOCS) 1 in T-cells which inhibits Th1 differentiation [26]. Hence, the presence of IL-6 may shift the Th1/Th2 balance towards Th2 [25]. On the other hand, IL-6 has been reported to act as a pro-inflammatory cytokine in several models of acute colitis. A neutralizing antibody against IL-6 receptor prevents colitis in TNBS colitis and in *IL-10^−/−^* mice [27] as well as in T-cell transfer colitis [28]. *IL-6^−/−^*-mice are reported to be less susceptible to DSS colitis [29].

In contrast, priming with *B. vulgatus* mpk *ex vivo* is known to induce semi-mature DC that show partial, IL-6-dependent resistance to a subsequent *E. coli* mpk stimulus [18]. The absence of colitis in *B. vulgatus* mpk mono-colonized *IL-2^−/−^* mice was associated with an increased expression of only IL-6 mRNA in the intestinal tissue [18]. Within the present study we were able to identify intestinal LP DC as one source of IL-6 in *B. vulgatus* mpk mono-colonized *IL-2^−/−^*-mice. Additionally, stimulation of DC with *B. vulgatus* mpk resulted in a decreased expression of CCR7 and led to a reduced migration of DC *in vitro*. This effect turned out to be IL-6 dependent, too. This is in line with recent studies reporting that DC pre-treated with IL-6 developed a semi-mature phenotype and downregulated CCR7 expression [19].

*B. vulgatus* mpk is phylogenetically closely related to *Porphyromonas gingivalis*. The LPS of *P. gingivalis* is less potential compared to enterobacterial LPS [30], [31]. Our group was able to show that the induction of semi-mature tolerogenic DC depended on the antigen concentration used for DC priming. Low antigen concentrations differentiated DC in dependency of IL-6 to tolerogenic DC that were non-responsive to subsequent TLR stimuli [20].

Which host pattern recognition receptors (PRR) mediate the interaction of luminal bacteria with the mucosa associated immune system is topic of ongoing discussions. Recently, it was shown that the induction of colitis in *IL-2^−/−^*-mice seems to be toll-like-receptor (TLR) independent, as *IL-2^−/−^ MyD88^−/−^ TRIF^−/−^* triple-deficient mice still developed colitis [32]. Whether in our model system TLRs or other PRRs have an impact on the IL-6 dependent modulation of DC by *B. vulgatus* mpk is topic of current experiments.

*B. vulgatus* mpk mono-colonized mice only showed expression of IL-6 mRNA [18], whereas *E. coli* mpk mono-colonized mice, prone to develop colitis and SPF *IL-2^−/−^-*mice already suffering from colitis, revealed high levels of IL-1α, IL-1β, TNF-α, IFN-γ, IL-10 and IL-6 mRNA in the intestine [3]. Therefore we hypothesize that under physiologic conditions IL-6 possibly contributes to maintenance of gut homeostasis, but in acute IBD with presence of pro-inflammatory cytokines IL-6 may act as a pro-inflammatory cytokine.

We found an increased CCR7 expression of MLN DC but not of LP DC in *E. coli* mpk mono-colonized mice prior to onset of colitis. This might be explained by the fact that upon activation DC upregulate CCR7 which confers responsiveness to CCL19 and CCL21 and migrate to secondary lymphoid organs [33], [34]. In *B. vulgatus* mpk mono-colonized and germfree mice, we found a low basic expression of CCR7 in both, LP DC and MLN DC indicating that there is constitutive trafficking of LP DC under steady-state conditions. This is in line with recent studies showing a constitutive trafficking of DC from the intestine to local lymph nodes in the absence of overt inflammation [35]. *B. vulgatus* mpk induced semi-mature IL-6 producing DC which showed only low constitutive trafficking, may differentiate adjacent LP DC to a semi-mature phenotype by a paracrine IL-6 loop. The local IL-6 cytokine milieu combined with the induction of semi-mature LP DC might inhibit T-cell activation and thereby inflammation as IL-6-STAT-3 mediated processes were shown to decrease the intracellular MHC-II αβ dimer, li (invariant chain) and H2-DM on DC and suppressed CD4^+^ T-cell mediated immune responses [24]. However, at present this hypothesis is highly speculative and further investigations have to elucidate the impact on *B. vulgatus* mpk primed IL-6 producing LP DC on the immune-homeostasis of the intestine.

In line with the increased CCR7 expression on MLN DC, *E. coli* mpk mono-colonized *IL-2^−/−^-*mice had a significantly diminished percentage of CD11c^+^ cells in the lamina propria as compared to *B. vulgatus* mpk mono-colonized mice. This might reflect the migration of mature LP DC of *E. coli* mpk mono-colonized mice to the MLN. The absolute LP DC number in SPF mice was enhanced, probably due to the severe inflammation in the intestine of these mice. Interestingly, we found an enhanced fraction of plasmacytoid DC (pDC) in SPF *IL-2^−/−^*-mice which already suffered from colitis as well as in *E. coli* mpk mono-colonized mice prone to develop colitis. Paradoxically, pDCs appear to have an intrinsic capacity to prime naive T-cells to differentiate into IL-10-producing T_reg_ cells at a mature stage [36], [37]. Further studies have to evaluate whether this represents a counter regulation of the intestinal immune system which is not effective in *IL-2^−/−^*-mice as these animals lack T_reg_ cells due to the IL-2 gene deficiency.

In summary, we conclude that mono-colonization of *IL-2^−/−^-*mice with *B. vulgatus* mpk drives LP DC to a semi-mature phenotype, characterized by reduced expression of DC activation markers and the DC homing factor CCR7. This protective effect of *B. vulgatus* mpk might be related to IL-6 secreted from *B. vulgatus* mpk primed LP DC. Therefore, our data provide new hints for an immune-regulating rather than immune-activating role of IL-6 in the colitis model of *IL-2^−/−^-*mice suggesting that IL-6 in the absence of TNF-α, IL-12 and IFN-γ exerts anti-inflammatory effects.

## Materials and Methods

### Animals and bacteria

Heterozygous mice from a mixed C57BL/6x129/Ola background were crossed to obtain *IL-2^−/−^* and wild type mice as described previously [3]. The mice were bred under gnotobiotic conditions at the University of Ulm, Germany. Gnotobiotic mice were maintained in a germfree environment or were mono-colonized with *E. coli* mpk [3] or *B. vulgatus* mpk [3]. The gnotobiotic state was controlled weekly and at the time of necropsy by culturing aerobic and anaerobic bacteria. At the age of 15 weeks mice were sacrificed. Animal experiments were reviewed and approved by an appropriate institutional review committee (Anzeige vom 01.05.06 Regierungspräsidium Tübingen).

### Isolation of lamina propria and MLN leukocytes

For isolation of MLN leukocytes mesenteric lymph nodes were taken from IL-2 mice and extruded through a mesh, washed twice and collected for analysis by flow cytometry.

For isolation of LP leukocytes, the small and large intestine was removed, sliced and washed in PBS to remove fecal content. To remove the epithelium, the tissue was incubated by gentle shaking in separation medium containing 1 mM dithiothreitol (DTT) and 1 mM EDTA in Ca^2+^/Mg^2+^-free PBS supplemented with 1% FCS for 15 min. To eliminate residual epithelial cells the tissue was washed in PBS containing 1% FCS and then cut into small pieces, resuspended in digestion medium consisting RPMI 1640, 5% FCS, 0.5 mg/ml collagenase type VIII (Sigma) and 5 U/ml DNase (Roche) and incubated for 90 min at 37°C by gentle shaking. Cells were passed through a mesh, centrifuged and the pellet was resuspended in 40% Percoll and carefully overlaid onto 70% Percoll. The Percoll gradient was centrifuged for 20 min at 2000 rpm and the interface containing the lamina propria leukocytes was collected and washed in PBS with 1% FCS. The cell number was determined and the cells were subjected to flow cytometry analysis as described below [38].

### Isolation of BMDC

Bone marrow cells were isolated and cultured as described by Lutz et al. [39] with minor modifications. Cells were harvested at day 8 and used to evaluate the effects of cellular challenge with *E. coli* mpk or *B. vulgatus* mpk on migration and CCR7 expression as described below.

### Stimulation of BMDC with B. vulgatus mpk or E. coli mpk

Cells were stimulated at day 8 with viable bacteria MOI 10 at 37°C, 5% CO_2_. Gentamicin was added one hour after stimulation and cells were incubated for 4 h (mRNA expression analysis) or 20 h (flow cytometry analysis). After 20 h CFU of viable bacteria was determined to exclude bacterial overgrowth. Stimulation with PBS was used as mock control. To exclude effects of gentamicin the antibiotic was also added to the mock control.

### Flow cytometry

BMDC were stimulated with *B. vulgatus* mpk or *E. coli* mpk as described above. 0.5 µg mAb/1×10^6^ cells were incubated for 30 min at 4°C. 30 000 cells were analyzed by flow cytometry. LP DC were pre-incubated with the mAb 2.4G2 directed against the FcγRIII/II CD16/CD32 to block non-specific binding of antibodies to Fc-receptors. The following antibodies were used for staining: FITC conjugated anti-mouse CD40, clone 3/23 (rat IgG_2a_,κ), FITC conjugated anti-mouse CD3ε, clone 145-2C11 (Armenian Hamster IgG_1_, κ), FITC conjugated anti-mouse CD49b/Pan-NK cells, clone DX5 (rat IgM, κ), FITC conjugated anti-mouse CD19, clone 1D3 (rat IgG_2a_, κ), PE conjugated anti-mouse CD11c, clone HL3 (Armenian Hamster IgG_1_, λ), biotinylated anti-mouse CD80, clone 16-10A1 (Armenian Hamster IgG_2_, κ), biotinylated anti-mouse CD86, clone GL1 (rat IgG_2a_, κ), biotinylated anti-mouse I-A^b^, clone AF6-120.1 (mouse IgG_2a_, κ), biotinylated anti-mouse CD40, clone 3/23, biotinylated anti-mouse CCR7, clone 4B12 (rat IgG_2a_), APC conjugated anti-mouse CD45R/B220, clone RA3-6B2 (rat IgG_2a_, κ) and APC conjugated anti-mouse CD11b, clone M1/70 (rat IgG_2b_, κ). SAv-PerCP and SAv-APC were used to detect binding of biotinylated antibodies.

### Intracellular cytokine staining

LP T-cells from *IL-2^−/−^*-mice were isolated as described above and stimulated with paramethoxy-amphetamine (PMA, Sigma), Ionomycin (Sigma) and Monesin (BD Pharmingen) for 3 h and stained with FITC conjugated anti-mouse CD3ε, clone 145-2C11 (Armenian Hamster IgG_1_, κ) and APC conjugated anti-mouse CD4, clone RM4-5 (rat IgG_2a_, κ). As the absolute number of lamina propria DC isolated from the intestine of non-inflamed *B. vulgatus* mpk mono-colonized mice was too small to perform FACS sorting, RNA analysis and intracellular cytokine staining in DC, the *IL-2^−/−^*-mice received at the age of 13 weeks s.c. 1×10^6^ B16-FL cells, a murine melanoma tumor cell line engineered to stably produce murine Flt3-L [40]. The mice were killed after 14 days. The expression of activation and maturation markers was unchanged as compared to untreated mice (Figure S6). Isolated LP leukocytes were stained with APC conjugated anti-mouse CD11c, clone HL3 (Armenian Hamster IgG_1_, λ2).

For intracellular staining cells were fixed and permeabilized with 150 µl BD Cytofix/Cytoperm™ for 20 min at 4°C. After washing twice with BD Perm/Wash™ cells were stained for 30 min at 4°C in 50 µl BD Perm/Wash™ (concentration: 0.5 µg mAb/1×10^6^ cells). For T-cells PE conjugated anti-mouse IFN-γ, clone XMG1.2 (rat IgG_1_), PE conjugated anti-mouse TNF-α, clone MP6-XT22 (rat IgG_1_), PE conjugated anti-mouse IL-17, clone TC11-18H10.1 (rat IgG_1_, κ), PE conjugated anti-mouse IL-10, clone JES5-16E3 (rat IgG_2a_) and PE conjugated anti-mouse IL-4, clone 11B11 (rat IgG_1_) were used. DC were stained with PE conjugated anti-mouse IL-6, clone MP5-20F3 (rat IgG_1_) or with PE conjugated anti-mouse TNF-α, clone MP6-XT22 (rat IgG_1_). Afterwards, cells were washed twice with BD Perm/Wash™ and resuspended in PBS with 1% FCS prior to flow cytometric analysis.

CD11c^+^ cell sorting was performed by FACSAria. The collected cells were resuspended in RLT-Buffer for subsequent analysis of IL-6 and TNF-α mRNA expression.

### Determination of CCR7 and IL-6 mRNA expression in BMDC or LP DC by quantitative real-time RT-PCR (qRT-PCR)

3×10^6^ BMDC were stimulated with *E. coli* mpk or *B. vulgatus* mpk for 4 h as described above. LP DC were isolated from the intestine of *E. coli* mpk or *B. vulgatus* mpk mono-colonized *IL-2^−/−^*-mice, respectively. After stimulation or after FACS sorting of CD11c^+^ cells, RNA isolation was performed according to the manufacturer's instructions (Quiagen RNeasy Mini Kit 250). Extracted RNA was dissolved in water containing 0.1% diethyl-pyrocarbonate (DEPC). For reverse transcription 4 µg of RNA were mixed with 0.5 µg oligo (dT)_ 12-18_ primer (Invitrogen Life Technologies) and DEPC water was added to a final volume of 10 µl, followed by incubation at 65°C for 10 min. After adding 10 µl of a solution containing 5× first strand buffer, 20 nmol/l DTT, 200 U Superscript II (Invitrogen Life Technologies), 40 U RNase Out (Invitrogen Life Technologies) and 2 mmol/l desoxynucleoside triphosphates (dNTPs) (Roth), the mixture was incubated at 37°C for 60 min. Finally the samples were heated at 90°C for 5 min, diluted with DEPC treated water, and stored at −20°C until further use.

qRT-PCR was carried out in duplicates in 96-well format on a GeneAmp 5700 Sequence Detection System (Applied Biosystems/Applera, Darmstadt, Germany). Each 20 µl reaction contained 10 µl TaqMan Universal PCR MasterMix (No AmpErase UNG, Applied Biosystems), 1 µl target gene specific Assay-on-Demand Gene Expression Assay Mix (Applied Biosystems), 4 µl PCR grade water and 5 µl cDNA. Thermal cycling conditions for all reactions were as follows: 2 min at 50°C, 10 min at 95°C, 40 cycles for 15 s at 95°C and 1 min at 60°C. The standard curve method was used for semiquantitative data analysis. Data were normalized by dividing the values of the target gene by the values of the housekeeping gene *gapdh*.

### Cell migration assay

On day 9 BMDC stimulated with *E. coli* mpk or *B. vulgatus* mpk were resuspended in RPMI 1640, washed and 5×10^5^ cells were added on the top of a transwell culture insert with 6.5 mm diameter and 3 µm pore size (BD Pharmingen). The chemokines CCL19 (100 ng/ml) and CCL21 (100 ng/ml) (both from Pepro Tech) were added to the lower compartment. CCL19 and CCL21 are the sole ligands for CCR7. The number of cells migrated to the lower chamber within 4 h was determined by flow cytometry.

### Statistics

Statistical analyses were performed using ANOVA-One way followed by Dunnett's post test or the unpaired Student's t-test. P values<0.05 were considered significant. Error bars represent±SEM.

## Supporting Information

Figure S1 LP DC activation and maturation in germfree, gnotobiotic and SPF IL-2−/−-mice: LP DC were isolated from the total intestine of GF, B. vulgatus or E. coli mono-colonized, E. coli/B. vulgatus co-colonized and SPF IL-2−/− mice and analyzed for expression of CD80 and CD86 by flow cytometry. The results are representative for at least three animals which were analyzed separately. Mean and SD were calculated from those independent experiments. Numbers indicate the percentage of positive cells±SD. * p<0.05 compared to germfree and B. vulgatus mono-colonized IL-2−/− mice.
(1.14 MB TIF)

Figure S2 LP DC were isolated from the total intestine of GF, *B. vulgatus* mpk or *E. coli* mpk mono-colonized, *E. coli mpk/B. vulgatus* mpk co-colonized and SPF *IL-2^+/+^* WT mice and analyzed for expression of CD40, CD80, CD86 and MHC-II by flow cytometry. The results are representative for at least three animals which were analyzed separately. Mean and SD were calculated from those independent experiments. Numbers indicate the percentage of positive cells±SD. * p<0.05 compared to germfree and B. vulgatus mono-colonized *IL-2^−/−^* mice.
(2.07 MB TIF)

Figure S3 TNF-α and IL-6 production in wild type or *IL-6^−/−^* BMDC upon stimulation with *B. vulgatus* mpk or LPS or *B. vulgatus* and LPS. Secretion of TNF-α and IL-6 was determined by ELISA. Experiments were performed in duplicates, values represent means±SD of duplicates, results are representative for three independent experiments. * p<0.05 compared to LPS stimulated DC. ** p<0.05 compared to LPS and *B. vulgatus* mpk and LPS stimulated DC.
(7.20 MB TIF)

Figure S4 CCR7 mRNA levels were determined by qRT-PCR. Analysis of CCR7 mRNA expression was performed in duplicates, values represent means±SD of duplicates, results are representative for three independent experiments. ** p<0.05 compared to mock control.
(0.52 MB TIF)

Figure S5 Cryostat sections of distal ileum and colon of GF, *B. vulgatus* mpk or *E. coli* mpk mono-colonized or *E. coli mpk/B. vulgatus* mpk co-colonized *IL-2^−/−^*-mice, were incubated with fluorescence labeled anti-mouse CD11c mAb, nuclei were stained with DAPI. Scale bar represents 30 µm.
(12.73 MB TIF)

Figure S6 *IL-2^−/−^*-mice were treated s.c. with murine FLT-3L and sacrificed after 14 days. LP DC were isolated from the total intestine and analyzed for expression of CD40 and MHC-II by flow cytometry. The results are representative for at least three animals which were analyzed separately.
(6.56 MB TIF)
