# Supplementary figures and images for: Intestinal Colonization of IL-2 Deficient Mice with Non-Colitogenic B. vulgatus Prevents DC Maturation and T-Cell Polarization
Source: PLoS One. 2008 Jun 11;3(6):e2376. doi: 10.1371/journal.pone.0002376 (PMC2398772; doi:10.1371/journal.pone.0002376)

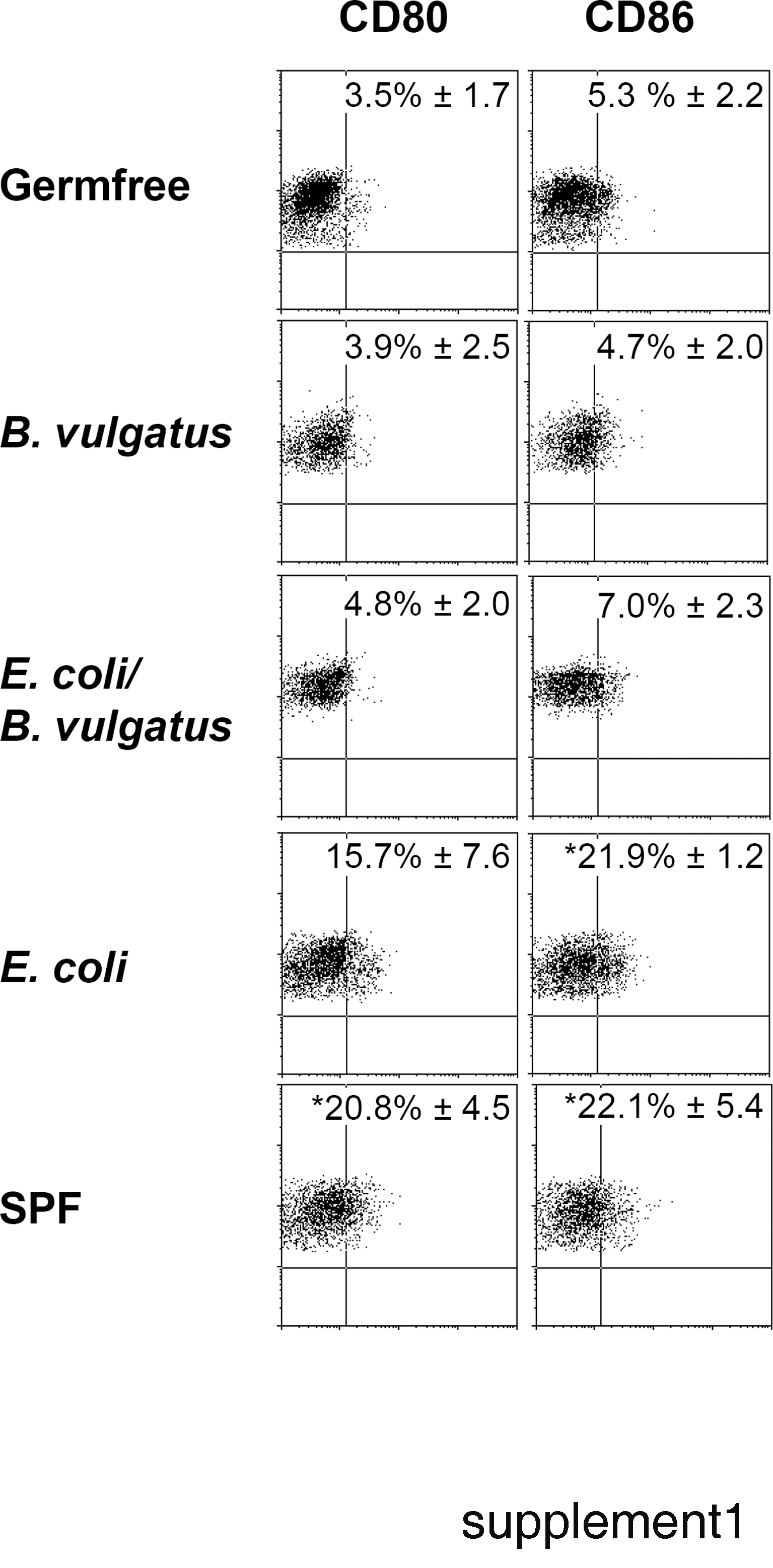

Supplement: Figure S1 — LP DC activation and maturation in germfree, gnotobiotic and SPF IL-2−/−-mice: LP DC were isolated from the total intestine of GF, B. vulgatus or E. coli mono-colonized, E. coli/B. vulgatus co-colonized and SPF IL-2−/− mice and analyzed for expression of CD80 and CD86 by flow cytometry. The results are representative for at least three animals which were analyzed separately. Mean and SD were calculated from those independent experiments. Numbers indicate the percentage of positive cells±SD. * p<0.05 compared to germfree and B. vulgatus mono-colonized IL-2−/− mice. (1.14 MB TIF) [file pone.0002376.s001.tif]

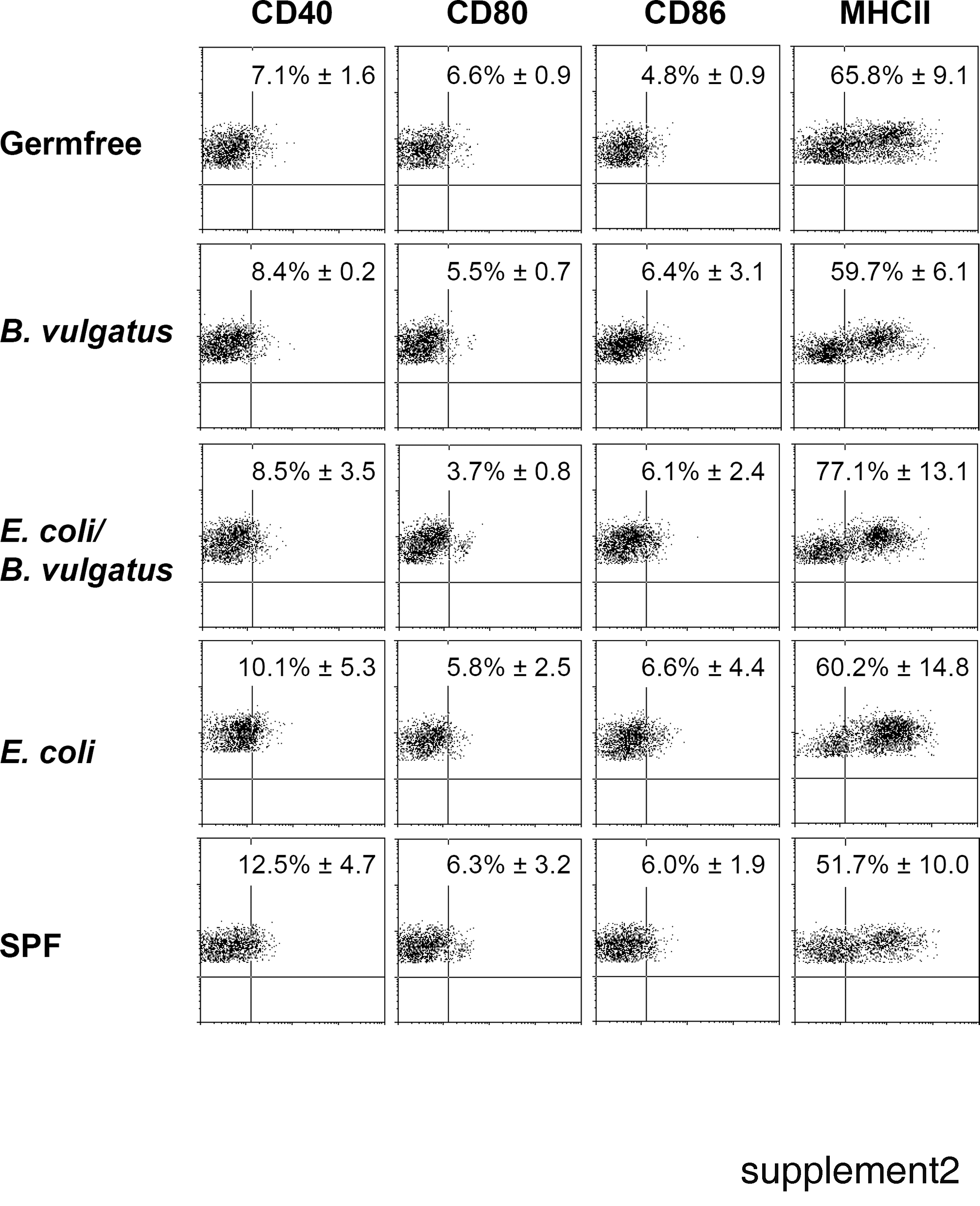

Supplement: Figure S2 — LP DC were isolated from the total intestine of GF, B. vulgatus mpk or E. coli mpk mono-colonized, E. coli mpk/B. vulgatus mpk co-colonized and SPF IL-2+/+ WT mice and analyzed for expression of CD40, CD80, CD86 and MHC-II by flow cytometry. The results are representative for at least three animals which were analyzed separately. Mean and SD were calculated from those independent experiments. Numbers indicate the percentage of positive cells±SD. * p<0.05 compared to germfree and B. vulgatus mono-colonized IL-2−/− mice. (2.07 MB TIF) [file pone.0002376.s002.tif]

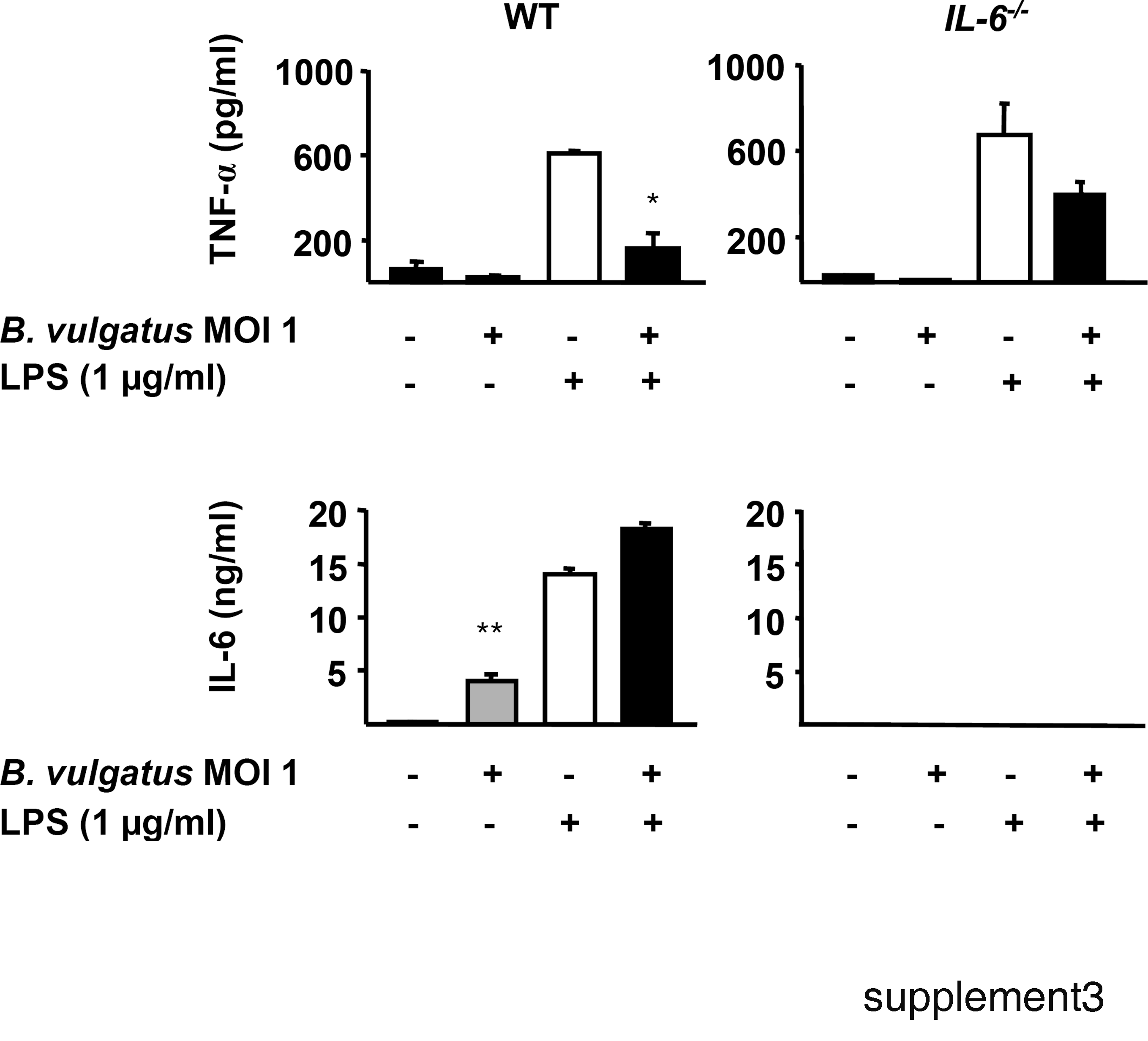

Supplement: Figure S3 — TNF-α and IL-6 production in wild type or IL-6−/− BMDC upon stimulation with B. vulgatus mpk or LPS or B. vulgatus and LPS. Secretion of TNF-α and IL-6 was determined by ELISA. Experiments were performed in duplicates, values represent means±SD of duplicates, results are representative for three independent experiments. * p<0.05 compared to LPS stimulated DC. ** p<0.05 compared to LPS and B. vulgatus mpk and LPS stimulated DC. (7.20 MB TIF) [file pone.0002376.s003.tif]

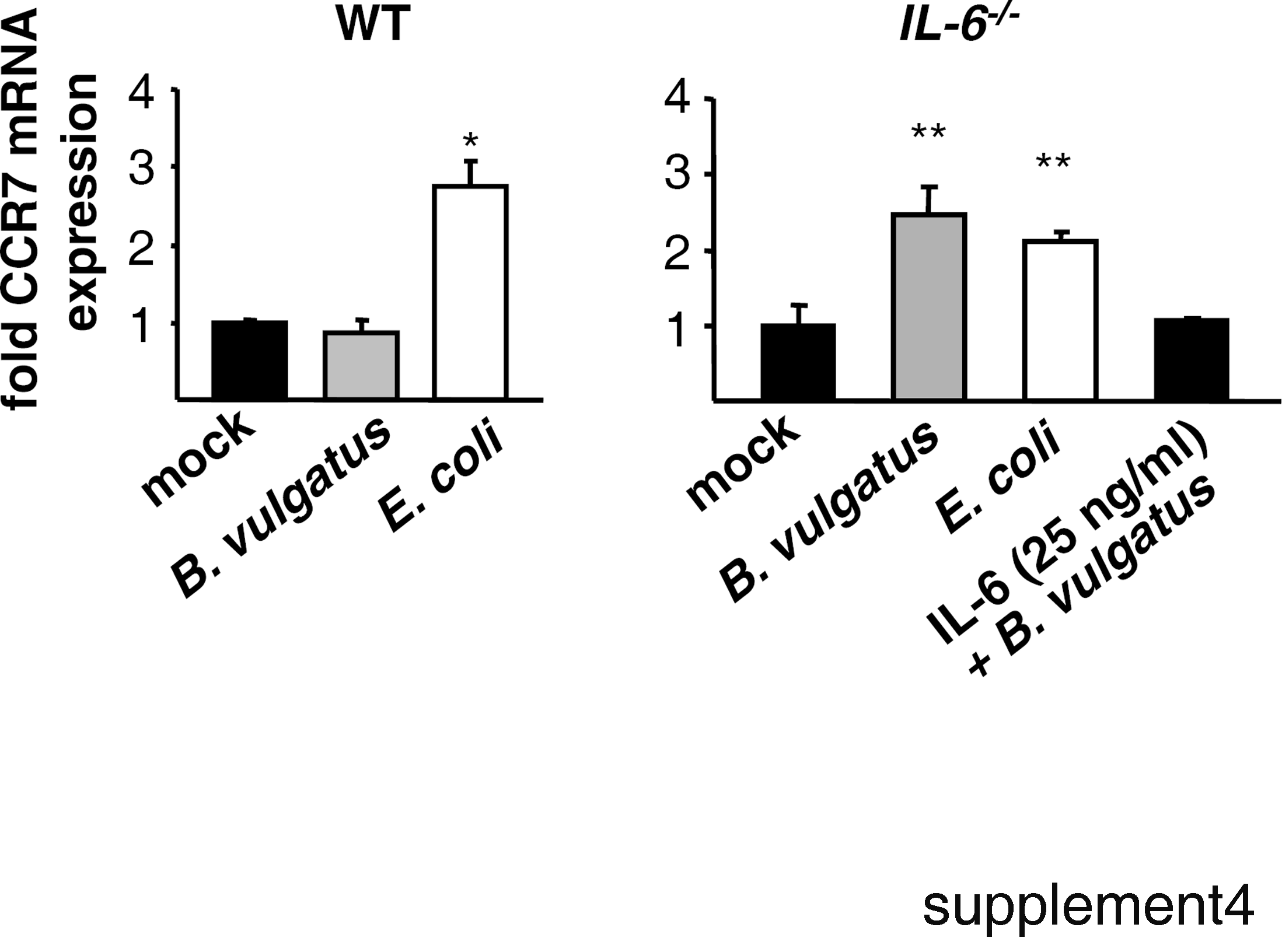

Supplement: Figure S4 — CCR7 mRNA levels were determined by qRT-PCR. Analysis of CCR7 mRNA expression was performed in duplicates, values represent means±SD of duplicates, results are representative for three independent experiments. ** p<0.05 compared to mock control. (0.52 MB TIF) [file pone.0002376.s004.tif]

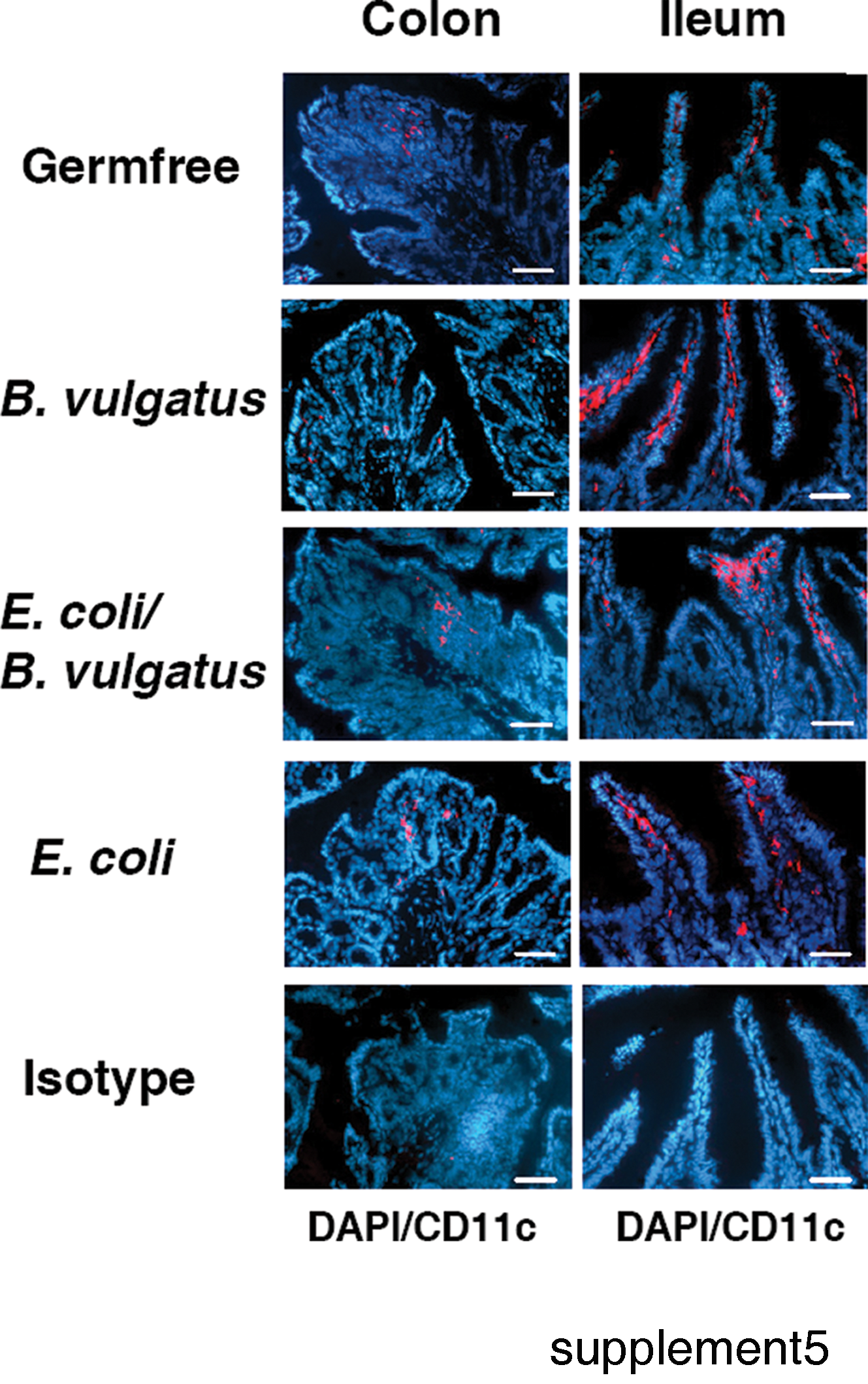

Supplement: Figure S5 — Cryostat sections of distal ileum and colon of GF, B. vulgatus mpk or E. coli mpk mono-colonized or E. coli mpk/B. vulgatus mpk co-colonized IL-2−/−-mice, were incubated with fluorescence labeled anti-mouse CD11c mAb, nuclei were stained with DAPI. Scale bar represents 30 µm. (12.73 MB TIF) [file pone.0002376.s005.tif]

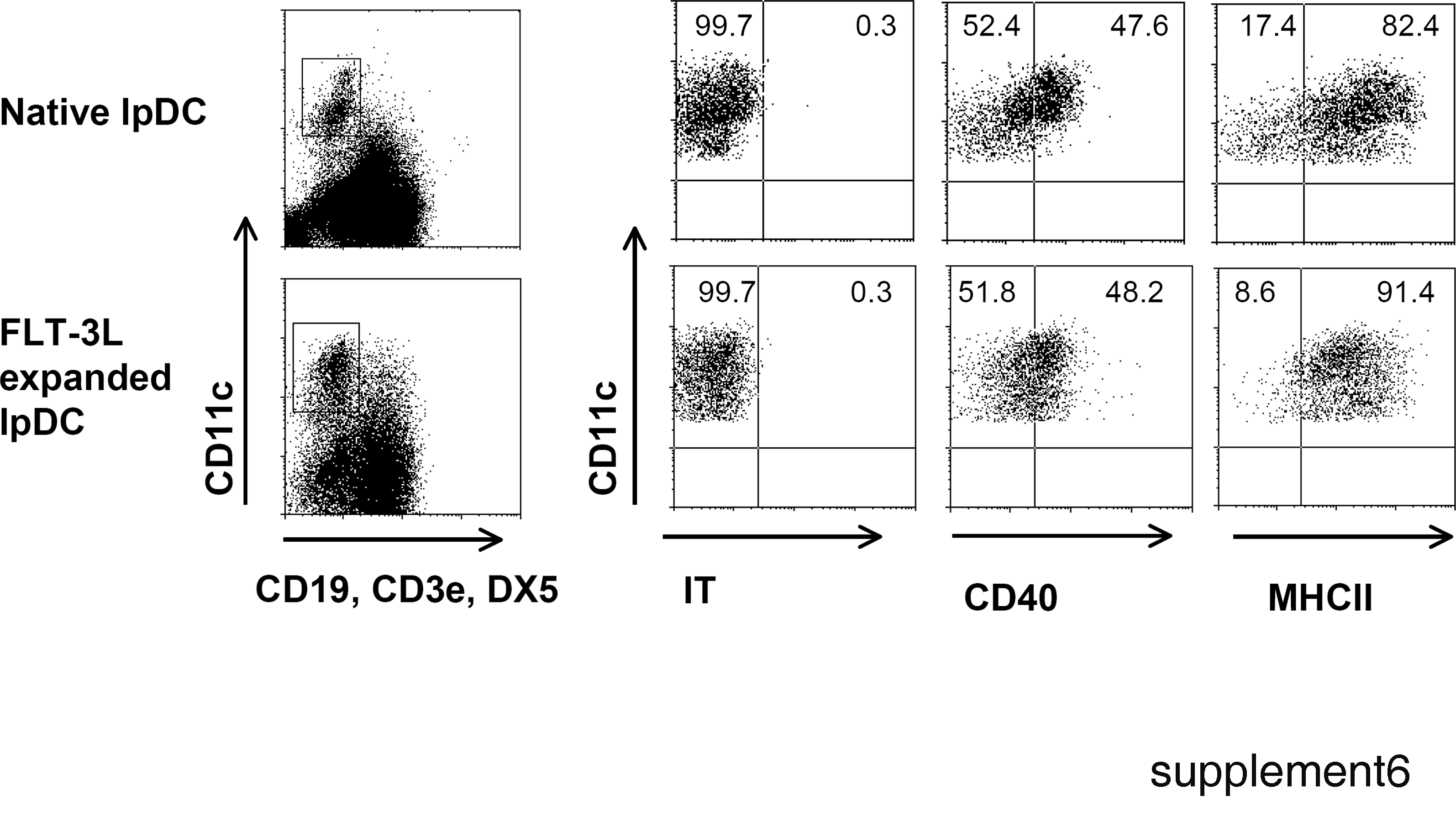

Supplement: Figure S6 — IL-2−/−-mice were treated s.c. with murine FLT-3L and sacrificed after 14 days. LP DC were isolated from the total intestine and analyzed for expression of CD40 and MHC-II by flow cytometry. The results are representative for at least three animals which were analyzed separately. (6.56 MB TIF) [file pone.0002376.s006.tif]
